# Current Prevention of COVID-19: Natural Products and Herbal Medicine

**DOI:** 10.3389/fphar.2020.588508

**Published:** 2020-10-16

**Authors:** Junqing Huang, Gabriel Tao, Jingwen Liu, Junming Cai, Zhongyu Huang, Jia-xu Chen

**Affiliations:** ^1^ Formula-pattern Research Center, School of Traditional Chinese Medicine, Jinan University, Guangzhou, China; ^2^ Department of Pharmacological and Pharmaceutical Sciences, College of Pharmacy, University of Houston, Houston, TX, United States; ^3^ Department of Biomedical Engineering, Henry Samueli School of Engineering, University of California, Irvine, Irvine, CA, United States; ^4^ School of Traditional Chinese Medicine, Beijing University of Chinese Medicine, Beijing, China

**Keywords:** coronavirus disease 2019, severe acute respiratory syndrome coronavirus 2, prevention, prophylactic, natural product, herbal medicine

## Abstract

Starting from December 2019, novel coronavirus disease 2019 (COVID-19) pandemic has caused tremendous economic loss and unprecedented health crisis across the globe. While the development of cure is at full speed, less attention and fewer effort have been spent on the prevention of this rapidly spreading respiratory infectious disease. Although so far, several vaccine candidates have advanced into clinical trials, limited data have been released regarding the vaccine efficacy and safety in human, not mention the long-term effectiveness of those vaccines remain as open question yet. Natural products and herbal medicines have been historically used for acute respiratory infection and generally show acceptable toxicity. The favorable stability for oral formulation and ease of scaling up manufacture make it ideal candidate for prophylactic. Hereby, we summarized the most recent advance in SARS-CoV-2 prevention including vaccine development as well as experimental prophylactics. Mainly, we reviewed the natural products showing inhibitory effect on human coronavirus, and discussed the herbal medicines lately used for COVID-19, especially focused on the herbal products already approved by regulatory agency with identifiable patent number. We demonstrated that to fill in the response gap between appropriate treatment and commercially available vaccine, repurposing natural products and herbal medicines as prophylactic will be a vigorous approach to stop or at least slow down SARS-CoV-2 transmission. In the interest of public health, this will lend health officials better control on the current pandemic.

## Introduction

The novel coronavirus disease 2019 (COVID-19) pandemic starting from December 2019 has cast unprecedented threat to public health worldwide with over 27.9 million infection cases and 905,000 death till September 10, 2020, and the case number is still soaring (Bai et al., 2020). COVID-19 is caused by severe acute respiratory syndrome coronavirus 2 (SARS-CoV-2), a member of beta-coronaviruses family (Walls et al., 2020). Coronaviruses (CoVs) are a family of large (ranging from 27–32 kb), enveloped, signal-strand positive-sense RNA viruses, which have characteristic club-like spikes on their surface (Walls et al., 2020). Currently, seven strains of human CoVs are reported. Four of them merely produce mild symptoms: Human coronavirus OC43 (HCoV-OC43), Human coronavirus HKU1 (HCoV-HKU1), Human coronavirus 229E (HCoV-229E), and Human coronavirus NL63 (HCoV-NL63), while the other three cause more severe symptoms: Middle East respiratory syndrome-related coronavirus (MERS-CoV), Severe acute respiratory syndrome coronavirus (SARS-CoV), Severe acute respiratory syndrome coronavirus 2 (SARS-CoV-2) (Su et al., 2016).

SARS-CoV-2 initiate its infection *via* the interaction with angiotensin-converting enzyme 2 (ACE2) receptors and transmembrane protease, serine 2 (TMPRSS2) on host cell membrane (Hoffmann et al., 2020; Zhang H. et al., 2020). The mortality of COVID-19 is mainly due to acute respiratory distress syndrome and severe cytokine release syndrome (Hirano and Murakami, 2020; Mehta et al., 2020). Although over hundreds of clinical trials and preclinical studies have been set to seek cures for tackling COVID-19, up to date there are no approved therapeutics for this widely spreading disease. As for the prevention of COVID-19, several vaccine candidates are in pipelines, but due to the insufficiency of clinical evidence the efficacy of those vaccines remains arguable yet. Also, the safety issue of vaccine involves adaptive immune response which is far more complicated than small-molecule drug toxicity. Moreover, the scale-up of vaccine production is challenging, given that most vaccines are nucleotide or protein-based products which require more delicate manufacturing and storage system compared to small molecules. Those features make it extraordinarily demanding to provide a validated COVID-19 vaccine in the short term.

Besides vaccine development, great efforts have been dedicated to discovering effective prophylactics against COVID-19 for high risk population, whereas very limited studies give satisfactory outcome. Very recently, several clinical cases and *in-vivo* results suggest that some anti-inflammation and anti-virus drugs have potential to be prophylactic candidates. But risk of adverse event will come along with the deployment of those medicines to big population, not to mention their preventive effect remains controversial. Natural products and herbal medicines have been used for the prevention of virus infection for years. Those medicinal products show favorable efficacy and tolerable toxicity. It is undeniable that herbal medicine is still a promising resource for drug discovery, and its acceptable toxicity make it a prospective prophylactic candidate against COVID-19. In face of this global health crisis, exploring prophylactics from herbal medicine is probably a promising and practical strategy to contain pandemic.

In this review, we aimed to provide a new perspective regarding COVID-19 prevention. We called attention to natural products and herbal medicines as potential prophylactic against COVID-19. We summarized the most recent advances in COVID-19 vaccine development and lately reported experimental prophylactics. Then, we discussed both the natural products inhibiting human coronavirus and the herbal medicines proven effective in alleviating respiratory distress syndrome. We performed integrated network analysis upon selected herbal medicine to identify the most promising active components with the potential to be prophylactics. Ultimately, this review attempts to offer alternative conceptual framework for COVID-19 prevention and deeper insight into this unprecedented pandemic.

## Current Prevention of COVID-19

Development of effective prevention is urged to contain the spread of current pandemic and halt the occurrence of future relapse. Vaccine, convalescent serum, monoclonal antibody, and marketed anti-viral drugs become the most promising options in the spotlight at this point. In this worldwide race to develop vaccine solution against SARS-COV-2, several candidates are standing out as the current front runners (**Table 1**) with the hope of deploying as early as the end of 2020. While vaccine development at full speed, repurposing or reinventing existing pharmaceutical solutions to meet the challenge will be also necessary. With established safety record, optimized mass-production infrastructure in place, repurposing could fast-track treatment options without compromising rigorous public health standards. To fill in the response gap between treatment and upcoming vaccine, repurposing preventative treatment, or prophylactics, could reduce transmission of the virus for the public and lend health officials better control on the outbreak. Several compounds of interests with ongoing trials are highlighted.

**Table 1:** COVID-19 vaccines in pipeline.

Lead Developer(s)	Vaccine Type	Development Status	Ref.
Moderna & NIAID	mRNA/nanoparticle	Phase III clinical trials ongoing	(Corbett et al., 2020; Moderna Inc, 2020)
AstraZeneca & University of Oxford	Plasmid edited gene materials/AdV	Phase I/II ongoing; phase II/III recruiting	(Oxford COVID-19 vaccine to begin phase II/III human trials | University of Oxford; Doremalen et al., 2020; Folegatti et al., 2020)
CanSino Biologics & Academy of Military Medical Sciences	Plasmid edited gene materials/Ad5	Phase I/II ongoing in China, phase I/II approved in Canada	(Halperin and Langley, 2020; Zhu et al., 2020; Zhu et al., 2020)
Sinovac Biotech & Wuhan Institute of Biological Products	Inactivated SARS-COV-2	Phase I/II ongoing	(Sinovac Biotech Co., Ltd, 2020)
Inovio Pharmaceuticals	SARS-COV-2 encoding DNA based	Phase I ongoing	(Inovio Pharmaceuticals, 2020; Smith et al., 2020)
BioNTech & Pfizer	mRNA vaccine	Phase II/III ongoing	(BioNTech SE, 2020; Mulligan et al., 2020a; Mulligan et al., 2020b)
Novavax	Recombinant Protein	Phase I complete, phase II ongoing	(Novavax Inc., 2020)
Johnson & Johnson	Recombinant Protein	Phase I/II ongoing, accelerating phase III	(Janssen Pharmaceutica, 2020)
Curevac	mRNA vaccine	Phase I ongoing	(CureVac Inc., 2020)
Imperial College London	RNA vaccine	Phase I/II ongoing	(ISRCTN - ISRCTN17072692: Clinical trial to assess the safety of a coronavirus vaccine in healthy men and women)
Russia Ministry of Health	Plasmid edited gene materials/AdV	Phase III ongoing, approved	(Logunov et al., 2020)

### Landscape of Global COVID-19 Vaccine Development

Most potential options can be broadly categorized three types: mRNA delivery, genetic material with viral carrier, and inactivated virus. Currently, more vaccines candidates on the most advanced timeline are in the second and third category for those methods have more responsive and rapid production scheme, suitable for emergent public health crisis of SARS-COV-2.

Two leading candidates on mRNA delivery are the mRNA-1273 by Moderna in collaboration with National Institute of Allergy and Infectious Diseases (NIAID) in the U.S., and BNT162b1 by BioNTech in collaboration with Pfizer in Germany. Both candidates use RNA motifs of encoding sequence for the Spike (S) protein. Since the commence of the vaccine, Moderna has announced FDA’s permission for phase III recruitment starting July 2020, implying expedited development is currently on track to delivery optimistically by late 2020 (Moderna Inc, 2020). As of early Septermber, Moderna’s phase III clinical trials has recruited more than 20,000 participants, on track with early estimate (ModernaTX. Inc, 2020). BioNTech, in similar timeline, is set to start phase III of clinical trial by the end of July 2020. To date, its global phase 2/3 trial has accumulatively enrolled more than 25,000 participants (BioNTech SE, 2020). It recently releases an interim report as preprint, showing positive response (~1.8–2.8 folds) to SARS-COV-2 when compared to convalescent human sera (Mulligan et al., 2020a). Under the class of genetic material with viral carrier, University of Oxford and Astra Zeneca have advance their candidate ChAdOx1 (AZD1222), a weakened adenovirus enclosure of SARS-COV-2 spike protein’s genetic material, into phase II/III clinical trial (Oxford COVID-19 vaccine to begin phase II/III human trials | University of Oxford). However, limited disclosed data are available in public. A similar and noteworthy candidate is from CanSino Biologics, in collaboration with Academy of Military Medical Sciences in China (Zhu et al., 2020). Both candidates use a variation of adenovirus, commonly found for common cold, are also immunogenic. This carrier approach has yet to be approved in the U.S. nor in EU previously. The other substantially competitive candidate is from Inovio Pharmaceuticals, whose candidate is a DNA based vaccine, INO-4800 (Smith et al., 2020). It announced a positive interim report on its phase I result, and has phase II/III trial expecting in August 2020 (Inovio Pharmaceuticals, 2020). Additionally, Russian Ministry of Health, in collaboration with Russian Direct Investment Fund (RDIF), has developed and approved its first vaccine Sputnik V, and later published its phase 1 results. The Russian vaccine is controversially fast-tracked in absence of phase III clinical trial process (Logunov et al., 2020). For inactivated virus, Sinovac from China is the leading candidate with its phase I/II trial ongoing in China and pending phase III trial in Brazil. This technology named PiCoVacc, uses inactivated or fragmented virus without replicability as the triggering cue to illicit patient’s sustained immunity, the ability to generate neutralizing antibody against SARS-COV-2 (Gao Q. et al., 2020).

Moreover, numerous contenders are collaborating with governments and philanthropic foundations to ramp up production to create stockpile once clinical trials provide evidence for their safety and efficacy, to shorten deployable timeline. NIAID also recently announce an establishment of a new clinical trial network focusing on COVID-19 vaccine and monoclonal antibody testing. However, mRNA vaccine, such as Moderna’s mRNA-1273, has yet to be approved and adopted historically. Lack of similar predecessor likely warrants more scrutiny from regulators and general publics.

### Convalescent Serum Transfusion and Monoclonal Antibody

Convalescent serum transfusion, where patient received recovered patients’ antibodies-contained plasma to develop the ability to fight viral infection, is currently widely trialed as treatment option for COVID-19 around the world. In April 2020, National Health Service (NHS) in the UK has established Blood and Transplant Service (NHSBT) to conduct recovery trial with Oxford University, studying the application viability of such technology on COVID treatment as well as prevention (NHS Blood and Transplant). To date, more than thousands of subjects are enrolled around the world in convalescent plasma clinical trials (Sheridan, 2020). FDA has also issue recommendation guideline for convalescent plasma investigational use (U.S. Food & Drug Administration, 2020). FDA has later issued an emergency use authorization (EUA) on August 23, while continued to encourage further clinical trials (U.S. Food & Drug Administration, 2020). Commercially, Regeneron has investigated antibodies purified human convalescent plasma and genetically humanized mice, in which the author shows an antibody cocktail of treatment potential (Hansen et al., 2020).

More recently, monoclonal antibody discovery against SARS-CoV-2 has yield valuable insight into variation of viable antibodies and their mechanisms. Wu et al. discussed the wide variation in neutralizing antibody (NAb) potency among infected patients, which was indicated with NAb titer (ID50: 200 ~ 21567) ranging across two orders of magnitudes (Wu F. et al., 2020). This highlight concerning needs for donor selection and post-processing effort (Robbiani et al., 2020). Another research group then provided example antibodies (B38 and H4) that binds with crucial target site like ACE2 and receptor-binding domain (RBD) (Wu Y. et al., 2020). Rogers et al. also revealed antibodies against both RBD and non-RBD epitopes of Spike (S) protein (Rogers et al., 2020). While Wang et al. identified 47D11 that has neutralization effect on both SARS-CoV and SARS-CoV-2, emphasized the domain conservation in SARS2-S-S1b (Wang C. et al., 2020). Zost et al. and Kreer et al. then add another collection of antibody candidate isolated from patients’ plasma. Moreover, Kreer et al. identified that the broad spectrum of variable genes shared among the potent B cells exists in naïve populations (Kreer et al., 2020; Zost et al., 2020). Both treatment and prophylactics based on antibody could likely be more immediate and rapidly available for high risk populations than a vaccine, but production ramping could introduce uncertainty for its timeline.

### Small Molecule Prophylactic

Remdesivir, developed by Gilead Sciences, acts as a prodrug of a nucleotide analog. When it is intracellularly metabolized, its product, analogue of adenosine triphosphate, will inhibit regular viral RNA polymerase’s function. It is considered applicable in board spectrum to multiple other coronaviruses, such as Ebola, MERS-COV, and SARS-COV. Remdesivir is not only propelled as a treatment against SARS-COV-2, but also is under investigation for prevention purpose. Early experiment of remdesivir by de Wit et al. (2020) shows both prophylactic and therapeutic benefits, mirroring the recent study on SARS-COV-2 on the same animal model (Casadevall and Pirofski, 2020). WHO is currently also studying remdesivir as one of the options for its “solidarity” clinical trial (World Health Organization, 2020).

Emtricitabine/tenofovir disoproxil, also commonly known for its brand name TRUVADA^®^ in the U.S., an HIV pre-exposure prophylactic. It is a combination of protease inhibitor and nucleotides reverse transcriptase inhibitor. It is considered to have beneficial effect on treating SARS-COV-2 with its possible inhibition of RNA-dependent RNA polymerase. A recent cohort study also suggests that AIDS patient taking antiretroviral therapy, especially tenofovir disoproxil fumarate/emtricitabine has lower risks of diagnosis, and reduced severity and fatality (del Amo et al., 2020). It is currently on track of the phase III clinical trial to prevent SARS-COV-2 high-risk healthcare workers in Spain, which sets to complete by August (Polo and Hernan, 2020).

Lopinavir/ritonavir, brand name KALETRA^®^ in the U.S., is a combination used as an HIV aspartate protease inhibitor previously, has *in vitro* testing inhibitory effect on SARS-COV-2 as well. However, it has not yet been observed efficacy in treating SARS-COV-2 severe patients (Cao et al., 2020). It was solicited by WHO for its SOLIDARITY clinical trial until July 2020. Recently, after NHS/Oxford study finds no clinical benefit from this combination’s usage, Lopinavir/ritonavir is discontinued onwards for WHO’s clinical trial recommendation (Horby et al., 2020).

## Natural Products Inhibiting Human Coronavirus

**Figure 1:**
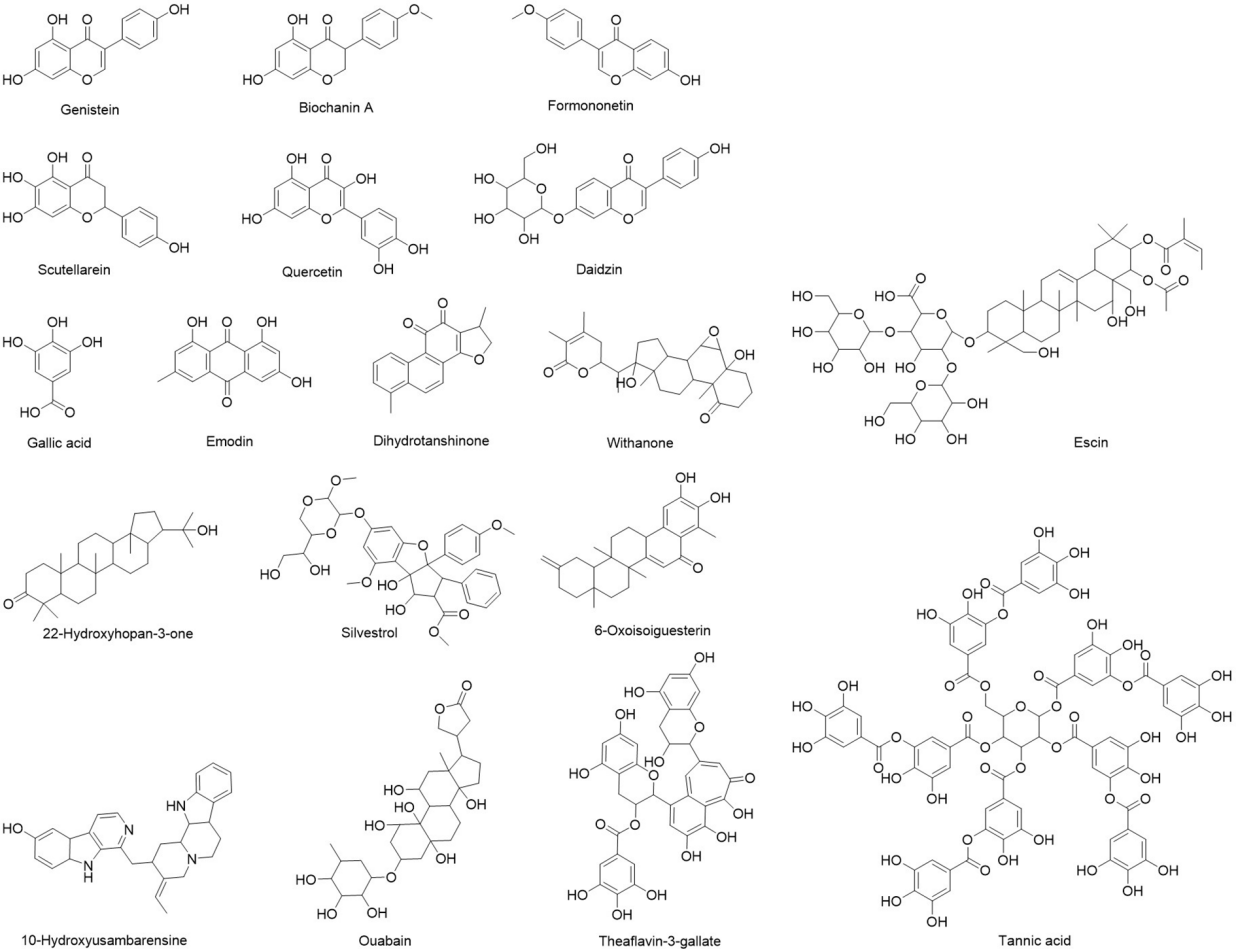
Natural products inhibiting human coronavirus.

Although combinatorial synthesis coupled with molecular docking help discover numerous synthetic drugs, more than one third of Food and Drug Administration (FDA)-approved drugs are natural products (Patridge et al., 2016). Plant, fungus and marine derived natural products have been rich resource of drug/nutrition discovery for many disease prevention (Tao et al., 2016; Wang et al., 2018; Lan et al., 2020). Natural products possess promising antiviral effects against human CoVs, which may guide the development of novel antiviral prophylactics. Here, we discussed updated researches focusing on natural products against MERS-CoV, SARS-CoV, or SARS-CoV-2 (**Figure 1**) and summarized their specific molecular targets and possible mechanisms of action (**Table 2**).

**Table 2:** Natural products potentially effective for COVID-19.

Natural Product	Inhibited Virus	Drug Targets/Relevant Signaling	Mechanism of Action	Ref.
Dihydrotanshinone	MERS-CoV	S protein of MERS-CoV	Block MERS-CoV entry using pre-and post-attachment assay	(Kim et al., 2018)
Ouabain	MERS-CoV	S protein of MERS-CoV	Block MERS-CoV entry by HCS assay, IC_50_ in Vero cells: 0.08 µM	(Ko et al., 2020)
Griffithsin	MERS-CoV	S protein of MERS-CoV	Inhibit spike protein function during entry	(Millet et al., 2016)
Silvestrol	MERS-CoV	eIF4A	Inhibit eIF4A, EC_50_: 1.3 nM	(Müller et al., 2018)
Emodin	SARS-CoV	S protein and ACE2 interaction	Blocked the binding of S protein to ACE2 using biotinylated ELISA assay, IC_50_: 200 μM	(Ho et al., 2007)
Scutellarein	SARS-CoV	SARS-CoV helicase protein	Inhibit the nsP13ATPase activity by FRET-based double-strand (ds) DNA unwinding assay, IC_50_: 0.86 ± 0.48 μM	(Yu et al., 2012)
Tannic acid	SARS-CoV	3CLPro	Inhibition of 3CLPro, IC_50_: 3 µM	(Chen et al., 2005)
Theaflavin-3-gallate	SARS-CoV	3CLPro	Blocking 3CLPro function, IC_50_: 7 µM	(Chen et al., 2005)
Escins	SARS-CoV	NF‐κB and activator protein-1 signaling pathways	Decrease levels of TNF‐α and IL‐6, EC_50_: 1.5 and 2.4 μg/ml in HCLE and NHC cells	(Michelini et al., 2018)
Daidzin	SARS-CoV-2	HSPA5	High binding affinity to HSPA5 SBDβ tested by virtual docking	(Elfiky, 2020)
Genistein				
Formononetin				
Biochanin A				
Lead compounds from *Alpinia officinarum*	SARS-CoV-2	PL protein	High binding affinity to PLpro tested by molecular docking	(Dibakar et al., 2020)
10-Hydroxyusambarensine	SARS-CoV-2	3CL protein	High binding affinity to 3CLpro tested by tested by molecular docking	(Gyebi et al., 2020)
6-Oxoisoiguesterin				
22-Hydroxyhopan-3-one				
Gallic acid	SARS-CoV-2	RdRp	High binding affinity to RdRp tested by molecular docking	(El-Aziz Abd et al., 2020)
Quercetin				
Withanone	SARS-CoV-2	TMPRSS2	Bind and interact at the catalytic site of TMPRSS2	(Kumar et al., 2020)

### Natural Products Inhibiting MERS-CoV

MERS-CoV causes Middle East respiratory syndrome (MERS, also known as camel flu). It was first discovered in 2012 in Saudi Arabia. Since then, it has spread to 27 countries through air travel of infected people (Sheahan et al., 2020), causing an outbreak of 2,494 cases and 858 deaths worldwide based on World Health Organization (WHO) report (WHO | Middle East respiratory syndrome coronavirus (MERS-CoV), 2020). MERS-CoV continues infecting human, thus it has been listed as a priority pathogen with pandemic potential by WHO. The mortality rate among patients with confirmed infection is approximately 37%. So far, very few studies have investigated the natural products as potential therapeutic agents for MERS-CoV.

The envelope spike (S) protein of MERS-CoV is important for dipeptidyl peptidase 4 receptor binding and virus-cell membrane fusion, thus it is the key for virus to entry host cells (Mille and Whittaker, 2014). Kim et al. (2018) generated a pseudo-virus expressing the S protein of MERS-CoV (MERS-PV) and screened 502 compounds derived from natural products to test their ability to block MERS-CoV entry. Three compounds (Dihydrotanshinone, E-64-C, and E-64-D) met the screening criteria at a concentration of 1 µg/ml. However, only dihydrotanshinone exhibits antiviral effects on MERS-CoV in the post-attachment assay. Dihydrotanshinone is extracted from the root of *Salvia miltiorrhiza* Bunge which is commonly used in traditional Chinese medicine. However, studies to confirm antiviral efficacy against MERS-CoV infection in animal model are required.

Ouabain is from the seeds of *Strophanthus gratus* (Wall. & Hook.) Baill. It has been used in cell biology studies as standard inhibitor of the Na^+^-K^+^-exchanging ATPase. A study (Ko et al., 2020) screened 5,406 compounds, including about 60% of all U.S. FDA-approved drugs, utilizing a Korean MERS patient isolate. By measuring the levels of the S protein expression of infected Vero cells using immunofluorescence analysis, they identified the cardiotonic drugs ouabain had a therapeutic index greater than 100, suggesting it could be considered for anti-MERS-CoV therapy. However, further *in vitro* and *in vivo* studies are needed to illustrate the mechanism of action.

Griffithsin isolated from the *Griffithsia* genus (red marine alga) is a 121 amino acid long lectin, which is attractive anti-coronavirus candidate because it interacts with coronavirus S proteins due to their highly glycosylated nature and represses coronavirus S protein functions (Millet et al., 2016). It has antiviral activity against HIV-1 within the picomolar range (EC_50_: 0.043 nM) (Mori et al., 2005). Milletit et al. have shown griffithsin is a potent inhibitor of MERS-CoV infection and production *in vitro* (Millet et al., 2016). In addition, Griffithsin has been shown a low systematic toxicity, hence making it a promising candidate against MERS-CoV.

Besides inhibiting MERS-CoV entry host cells, suppressing its replication is an alternative strategy. Silvestrol, a natural compound isolated from the plant *Aglaia foveolate* Pannell, is known for inhibition of the DEAD-box RNA helicase, eIF4A that participates in preparation of mRNA templates for ribosome recruitment during translation initiation (Todt et al., 2018). Thus, it suppresses the formation of virus replication. Müller and colleagues (Müller et al., 2018) investigated the inhibitory effects of silvestrol against MERS-CoV in human embryonic lung fibroblasts (MRC-5). Silvestrol was a potent antiviral molecule (EC_50_: 1.3 nM) with no major cytotoxic effects in the primary cells and in liver or spleen. For future studies, the antiviral effects of silvestrol need to be evaluated *in vivo* infection models to consolidate its therapeutic potential.

### Natural Products Inhibiting SARS-CoV

From 2002 to 2003, SARS-CoV emerged in Southern China, infecting more than 8,000 people and causing approximately 800 fatalities mostly in China and its neighboring countries. Like MRES-CoV, the envelope S protein of SARS-CoV is also essential for virus tropism and invasion into host cells, which is a potential target for the development therapeutics (Yuan et al., 2017). Angiotensin-converting enzyme 2 (ACE2) is identified as a functional receptor for SARS-CoV, which facilitates S protein-mediated infection, indicating it is also a possible target.

Emodin, an anthraquinone from *Rheum officinale* Baill and *Reynoutria multiflora* (Thunb.) Moldenke, has antibacterial and anti-inflammatory effects. Ho et al. (2007) have reported that emodin blocked the binding of S protein to ACE2 and reduced the infectivity of S protein pseudo-typed retrovirus to Vero E6 cells. Emodin effectively blocked the interaction between S protein and ACE2 in a dose-dependent manner with IC_50_ of 200 µM, indicating it might be a potential therapeutic agent for the treatment of SARS.

Scutellarein is a flavone found in *Scutellaria lateriflora* L. and other members of the genus *Scutellaria*. Yu et al. (2012) screened 64 purified natural compounds for the inhibitory effects of SARS helicase, nsP13 that possesses dsDNA unwinding activity and the ability to translocate along the nucleic acids, using fluorescence resonance energy transfer-based double-strand DNA unwinding assay. They found that scutellarein potently inhibited the SARS-CoV helicase protein *in vitro via* inhibiting the ATPase activity of nsP13. Scutellarein holds a promising potential for tackling SARS outbreaks; however, more preclinical/clinical studies to validate its efficacy are needed to evaluate their anti-viral effects.

Chymotrypsin-like protease (3CLPro) of SARS-CoV, an enzyme responsible for proteolysis, is vital to coronavirus replication, making it considered as an important target for drug discovery against SARS-CoV. Chen et al. (Doremalen et al., 2020) have screened a library with 720 compounds of natural product for inhibitory effect against 3CLPro of SARS-CoV by high-performance liquid chromatography assay and fluorogenic substrate peptide assay. Among them, two natural polyphenols found in black tea (*Camellia sinensis* (L.) Kuntze), tannic acid, IC_50_: 3 µM; theaflavin-3-gallate, IC_50_: 7 µM) showed desired benefits. Black tea is common all over the world, thus this study provides a new perspective that tea-derived supplements might prevent the infection of SARS-CoV. But detailed *in vitro* and *in vivo* studies need to be conducted, SARS-CoV infection is related to the release of pro-inflammatory cytokines and uncontrolled inflammation that induce the accumulation of intra‐alveolar fibrin and lead to pulmonary damage. Thus, an alternative strategy is to reduce inflammation. Escins are saponin mixtures from Japanese horse chestnut (seed of *Aesculus turbinata* Blume) that has been used as an herbal medicine. It has anti-inflammatory activities and anti-antiviral effects against SARS-CoV (an EC50 of 6.0 μM) (Wu et al., 2004). Escin has been reported to decrease the levels of TNF‐α and IL‐6 in J774A.1 cells infected with HSV-1 or stimulated with Toll‐like receptor ligands by the inhibition of NF‐κB and activator protein-1 signaling pathways (Michelini et al., 2018). However, the severe cytotoxic effects in human lung derived cells limits its potential to be a prophylactic. Usually, a more efficient or safer drug can be designed based on the original natural compound that exhibits the wanted activity. Kim et al. (2017) designed and synthesized a series of escin derivatives without the angeloyl or tigloyl groups that are important for cytotoxicity of escins and modified glycosidic linkages by hydrolysis. Those Escin derivative showed lower cytotoxicity.

### Natural Products Inhibiting SARS-CoV-2

The current COVID-19 pandemic caused by SARS-CoV-2 was identified in Wuhan City, in Hubei province of China. The number of infection case is still progressively growing. The genome of SARS-CoV-2 has over 70% similarity to that of SARS-CoV (Zhang and Holmes, 2020), leading to its current name. There is an urgent need to prevent and treat SARS-CoV-2 infection. Due to the high similarity, many approved or pre-clinical anti-SARS drugs are being tested for antiviral activity against SARS-CoV-2. For example, escin (Gallelli, 2020) and sodium aescinate injection (trial filed in China with ID : ChiCTR2000029742) have been registered. As natural products has been historically used for respiratory infection, there is arising voice calling for the repurposing of natural products for COVID-19 (Wang D. et al., 2020).

Heat Shock Protein A5 (HSPA5, also known as BiP or GRP78) is one of the host-cell receptors that have been reported to be recognized by virus S protein. When infected, HSPA5 is upregulated and translocated to the cell membrane where it is recognized by the SARS-CoV-2 spike to drive the infection process. Elfiky et al. (Elfiky, 2020) have tested several compounds from natural product against the HSPA5 substrate-binding domain β (SBDβ) by molecular docking and molecular dynamics simulations. The phytoestrogens (Daidzin, Genistein, Formononetin, and Biochanin A) and estrogens have proximal binding affinities with HSPA5. Those compounds may interfere with SARS-CoV-2 attachment to the host cells. Hopefully, those medicinal plants-derived compounds may guide the drug discovery in finding the suited prophylactics for SARS-CoV-2. Detailed studies investigating the antiviral bioactivity of those compounds should be further examined.

The host enzyme transmembrane protease serine 2 (TMPRSS2) facilitates viral particle entry into host cells. Inhibiting of this enzyme blocks virus fusion with ACE2, making it a potential target to inhibit virus entry. By molecular docking and molecular dynamics simulations, Kumar et al. (2020) have shown that withanone derived from Ashwagandha leaves (*Withania somnifera* (L.) Dunal) could bind and stably interact at the catalytic site of TMPRSS2 (His296, Asp345 and Ser441). In addition, they have confirmed that withanone significantly downregulated TMPRSS2 in MCF-7 cells, suggesting its dual potential to ramp down TMPRSS2 function.

SARS-CoV-2 papain-like protease (PL pro) cleaves the viral polyproteins a/b which is essential for its survival and replication. Thus, PL pro is one of the prospective drug targets of SARS-CoV-2. Goswami et al. (Dibakar et al., 2020) established a library of small molecules found in rhizomes, Alpinia officinarum (*Alpinia officinarum* Hance), ginger (*Zingiber officinale* Roscoe), and curcuma (*Curcuma longa* L.). The compounds were docked into the solvent accessible S3-S4 pocket of PLpro. In silico results showed eight lead compounds from galangal (*Alpinia officinarum* Hance) and ginger (*Zingiber officinale* Roscoe) bound with high affinity to SARS-CoV-2 PLpro, suggesting their potential as inhibitors against SARS-CoV-2. However, subsequent *in vitro* and *in vivo* experiments are needed to elucidate their efficacy against SARS-CoV-2.

Besides S protein and PLpro, the other promising drug target for combating the infection of SARS-CoV-2 is 3-chymotrypsin-like protease (3CLpro, also known as main protease). The conserved 3CLpro controls virus replication. Gyebi et al. (2020) have screened a series of alkaloids and terpenoids derived from African plants as potential inhibitors of 3CLpro using molecular docking and absorption, distribution, metabolism, excretion, and toxicity (ADMET) virtual analysis by the SuperPred webserver. The results revealed that 10-Hydroxyusambarensine, Cryptoquindoline, 6-Oxoisoiguesterin, and 22-Hydroxyhopan-3-one might be potent inhibitors with greatest drug-likeness against SARS-CoV-2 3CLpro.

RNA-dependent RNA polymerase (RdRp) is an essential virus replicase that catalyzes the synthesis of complementary RNA strands using the virus RNA template. The molecular structure of RdRp was revealed in May 2020 (Gao Y. et al., 2020), providing a new strategy for discovering prophylactic candidates for SARS-CoV-2 inhibition. Abd El-Aziz et al. (El-Aziz Abd et al., 2020) investigated the potential of eight natural polyphenols (quercetin, naringenin, caffeine, oleuropein, ellagic acid, benzoic acid, resveratrol, and gallic acid polyphenols) as inhibitors of SARS-CoV-2 RdRp by molecular docking assay. The studied polyphenols formed hydrogen bonds with the nucleotide triphosphate (NTP) entry channel amino acids (ARG 555, ARG 555, LYS 545) in SARS-CoV-2 RdRp (except caffeine and oleuropein). Binding to NTP may inhibit the entry of the substrate and subsequently repress the enzyme activity. The results suggested that gallic acid and quercetin exhibited high binding affinity to RdRp. The NSP12 is an important RdRp for the coronavirus replicative machinery, which binds to co-factors NSP7 and NSP8 to activate its ability to replicate long RNA. A recent study (Ruan et al., 2020) has established two homologous models for virtual screening. Cepharanthine, an alkaloid tetrandrine isolated from Stephania (*Stephania tetrandra* S.Moore), has been reported to have anti-inflammatory and antioxidant activities (Weber and Opatz, 2019). The study has shown Cepharanthine could bind to the interface active pockets of the SARS-CoV-2 NSP12-NSP8, suggesting it has therapeutic potential.

The researches mentioned above are all still in preliminary stages of drug development although they have shown great potentials against SARS-CoV-2 using computer-based screening. Further pre-clinical studies have to be performed to examine the anti-viral effects of those lead compounds. In the meanwhile, great number of clinical trials have registered to investigate the potentials of natural product to halt disease progression. For example, Koshak et al. from King Abdulaziz University will investigate the effects of Nigella sativa seed oil with immunomodulation and antiviral activity in hospitalized adult patients diagnosed with COVID-19 (Koshak et al., 2020). Corrao et al. from University of Palermo is recruiting patients to study the effectiveness of vitamin C to reduce mortality in patients (Corrao, 2020). Since the situation is uncontrollably worsening, many studies are being conducted on this topic, which may contribute to the rapid development of new prophylactics for COVID-19.

## Herbal Medicines Alleviating Acute Respiratory Infection

Herbal medicines like EPs^®^ 7630, Sinupret^®^, and KanJang^®^ have proven track record of treating acute respiratory infection due to common cold or influenza (Narimanian et al., 2005; Glatthaar-Saalmüller et al., 2011; Michaelis et al., 2011). Dating back to the beginning of COVID-19 outbreak around December 2019, herbal medicines were widely deployed across China to slow down the surge of infection cases. Its efficacy in alleviating acute respiratory distress syndrome caused by SARS-CoV-2 has been endorsed by both Chinese regulatory agency and the healthcare workers on the frontline. Recent perspectives from academics argued that the potential of herbal medicine to be an appropriate therapy for COVID-19 was open to question in the context that the pharmacological mechanism of those herbs remains unclear and hard to be fully explored (Gray and Belessis, 2020; Tao et al., 2020). That said, it is still undeniable that empirical therapy of herbal medicines contributed to the successful arrest of COVID-19 spreading in China to some extent, based on clinical observation. In addition, several preclinical studies lately proved that herbal medicines rich in flavonoid compounds had anti-virus activity in some human lung derived cell lines (Ding et al., 2017; Kong et al., 2020; Runfeng et al., 2020). Hereby, research digging into underlying mechanisms and identifying active components are urgently needed for the development of more effective herbal therapy and prophylactic.

A clinical case went public in March 2020 showed that a herbal formulation recommended by National Health Commission of the P.R. China (NHC) was effective in attenuating acute respiratory distress syndrome in a mild COVID-19 patient (Xu and Zhang, 2020). It was the first-of-its-kind to report the potential benefit of herbs in treating COVID-19. More recently several reviews systemically summarized the herbal medicines frequently used in China during COVID-19 pandemic and performed meta-analysis to illustrate its therapeutic outcome (Zhang D. et al., 2020). Xiong et al. indicated that among those herbal medicines widely distributed, Liquoric Root (*Glycyrrhiza glabra* L.), Baical Skullcap Root (*Scutellaria baicalensis* Georgi), Pinellia Rhizome [*Pinellia ternata* (Thunb.) Makino], Forsythia Fruit [*Forsythia suspensa* (Thunb.) Vahl], and Bitter Apricot Seed (*Prunus armeniaca* L.) are the most frequently prescribed herbs (Li et al., 2020). Their meta-analysis showed that herbal medicines are effective in halting the disease progression from mild to critical, decreasing hospitalization rate, shortening time of hospital stay, as well as alleviating COVID-19 associated symptoms like fever, cough, fatigue, and inflammation (Li et al., 2020).

Li et al. tested the potency of Lian-Hua-Qing-Wen, a licensed herbal formulation in inhibiting SARS-CoV-2 infection of Vero E6 cells using cytopathic effect inhibition assay and plaque reduction assay (Runfeng et al., 2020). The results showed that Lian-Hua-Qing-Wen significantly inhibited SARS-CoV-2 replication in Vero E6 cells and reduced pro-inflammatory cytokines like TNF-α, IL-6, CCL-2/MCP-1, and CXCL-10/IP-10 at mRNA level. Though its IC_50_ with over 400 μg/ml is a far cry from remdesivir efficacy, this study inspires that this herbal formulation can be validated as ideal prophylactic considering its moderate toxicity. Yang et al. performed LC-MS/MS and integrated network analysis to identify the active components of Qing-Fei-Pai-Du and Ma-Xin-Shi-Gan and their possible mechanism of action (Yang et al., 2020). The study revealed great number of compounds making up these two herbal formulations. Those chemicals mainly fall into four categories: flavonoids, glycosides, carboxylic acids, and saponins. In particular, glycyrrhizic acid isolated from Ma-Xin-Shi-Gan exhibited its anti-inflammatory effect by blocking toll-like-receptor and suppressing IL-6 production in macrophage. Huang et al. identified that quercetin, kaempferol, luteolin, isorhamnetin, baicalein, naringenin, and wogonin are probably the main active compounds responsible for the potency of herbs (Huang et al., 2020). Through *in silico* study, they hypothesized that ACE2, 3CL protein as well as intracellular signaling composed of COX-2, CASP3, MAPK, arachidonic acid, HIF-1, NF-κB, and Ras are all potential targets of herbal medicines. Most recently Ma et al. reported that Liu-Shen a herbal formulation exhibit favorable inhibitory effect against SARS-CoV-2 replication and virus-induced inflammation *in vitro* probably *via* suppressing NF-κB pathway (Ma et al., 2020).

**Figure 2:**
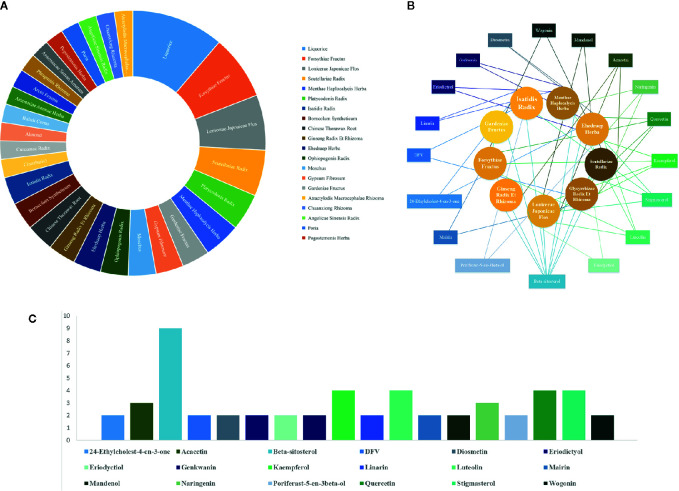
Chinese herbal medicines alleviating acute respiratory infection **(A)** ranking of main components in selected herbal medicines according to prescription frequency, **(B)** integrated network analysis of herbal components and lead compounds, **(C)** incidence of lead compounds occurring in principal herbal components.

Based on recently emerging studies, we reviewed all the herbal products both used for COVID-19 and approved by regulatory agency of which the patent numbers are identifiable. We summarized the composition and prospective drug targets of those licensed herbal products (**Table 3**) through literature search. We then ranked their ingredients based on dosage and frequency of use (**Figure 2A**). By narrowing down the sample volume, top nine herbal ingredients were identified as following: Glycyrrhizae Radix Et Rhizoma (*Glycyrrhiza inflata* Batalin), Forsythiae Fructus [*Forsythia suspensa* (Thunb.) Vahl], Lonicerae Japonicae Flos (*Lonicera Japonica* Thunb.), Scutellariae Radix *(Scutellaria baicalensis* Georgi), Platycodonis Radix [*Platycodon grandiflorum* (Jacq.) A. DC.], Menthae Haplocalycis Herba (*Mentha canadensis* L.), Gardeniae Fructus (*Gardenia jasminoides* J.Ellis), Gypsum Fibrosum, and Moschus (*Moschus anhuiensis*). Furthermore, we performed integrated network analysis upon those top nine herbal ingredients to identify eighteen lead compounds with greatest drug-likeness potential (**Figures 2B, C**). It showed that ten of those compounds are flavonoid derivatives which possess either flavone or flavanone core. It is known that many flavonoid compounds exhibit a broad spectrum of biological activities including cell membrane protective function, antioxidant activity *via* inhibition of xanthine oxidase or nitric oxide synthase as well as anti-inflammatory activity *via* inhibition of leukotriene (Verma and Pratap, 2010). Based on our integrated network analysis, COX-2 and MAPK mediated inflammatory pathways play prominent role in the therapeutic effect of these eighteen herb-derived compounds. Since many of those compounds have been historically used in dietary supplements, its toxicity is largely negligible, which makes them safe to be employed as prophylaxis against COVID-19 for large population.

**Table 3:** Licensed Chinese herbal medicines for acute respiratory infection.

Herbal Medicines	Affected Pathways Potential Targets	Composition		Ref.
		Herbal components	Original Species	
Lian-Hua-Qing-Wen	MAPK8, IL-6, COX-2, sEH, RELA, cPLA2α, mPGES-1, TNF, DPP4, IL-1β, CASP3, MAPK1, EGFR, BAX, BCL2, JUN, PIK3CG.	Forsythiae Fructus	*Forsythia suspensa* (Thunb.) Vahl	(Runfeng et al., 2020; Zhang D. et al., 2020)
		Lonicerae Japonicae Flos	*Lonicera Japonica* Thunb.
		Ehedraep Herba	*Ephedra sinica* Stapf
		Armeniacae Seman Amarum	*Prunus armeniaca* L.
		Gypsum Fibrosum^†^	
		Isatidis Radix	*Isatis tinctoria* L.
		Dryopteridis Crassirhizomatis Rhizoma	*Dryopteris crassirhizoma* Nakai
		Houttuyniae Herba	*Houttuynia cordata* Thunb.
		Pogostemonis Herba	*Pogostemon cablin* (Blanco) Benth.
		Rhei Radix Et Rhizoma	*Rheum palmatum* L.
		Rhodiolae Crenulatae Radix Et Rhizoma	*Rhodiola crenulata* (Hook.f. & Thomson) H.Ohba
		Menthol	*Mentha × piperita* L.
		Glycyrrhizae Radix Et Rhizoma	*Glycyrrhiza inflata* Batalin
Huo-Xiang-Zheng-Qi	PTGS2, HSP90AB1, mPGES-1, LTA4H, NOS2, PTGS2.	Atractylodis Rhizoma	*Atractylodes lancea* (Thunb.) DC.	(Zhang D. et al., 2020)
		Citri Reticulatae Pericarpium	*Citrus × aurantium* L.
		Magnoliae Officinalis Cortex	*Magnolia officinalis* Rehder & E.H.Wilson
		Angelicae Dahuricae Radix	*Angelica dahurica* (Hoffm.) Benth. & Hook.f. ex Franch. & Sav.
		Poria mushroom^§^	*Poria cocos* (Schw.) Wolf
		Arecae Pericarpium	*Areca catechu* L.
		Pinelliae Rhizoma	*Pinellia ternate* (Thunb.) Makino
		Glycyrrhizae Radix Et Rhizoma	*Glycyrrhiza inflata* Batalin
		Pogostemonis Herba	*Pogostemon cablin* (Blanco) Benth.
		Perillae Folium	*Perilla frutescens* (L.) Brittonon
Jin-Hua-Qing-Gan	COX-2, sEH, 5-LOX, PTGS2, AKTI, HSP90AA1, RELA, MAPK1, CASP3, TP53, ALB, TNF, IL6, MAPK8, MAPK14.	Lonicerae Japonicae Flos	*Lonicera Japonica* Thunb.	(Runfeng et al., 2020; Zhang D. et al., 2020)
		Gypsum Fibrosum^†^	
		Ehedraep Herba	*Ephedra sinica* Stapf
		Armeniacae Seman Amarum	*Prunus armeniaca* L.
		Scutellariae Radix	*Scutellaria baicalensis* Georgi
		Forsythiae Fructus	*Forsythia suspensa* (Thunb.) Vahl
		Fritillaria Thunbergii Bulbus	*Fritillaria thunbergii* Miq.
		Anemarrhenae Rhizoma	*Anemarrhena asphodeloides* Bunge
		Arctii Fructus	*Arctium lappa* L.
		Artemisiae Annuae Herba	*Artemisia annua* L.
		Menthae Haplocalycis Herba	*Mentha canadensis* L.
		Glycyrrhizae Radix Et Rhizoma	*Glycyrrhiza inflata* Batalin
Shu-Feng-Jie-Du	IL6, IL1B, CCL2, IL2, MAPK8, MAPK1, MAPK14, CASP3, FOS, ALB, IL4, IL1B, EGFR, FOS, AR, BCL2L, NOS2, F10, PTGS2, PTGS1, ESR1, DPP4.	Polygoni Cuspidati Rhizoma	*Reynoutria japonica* Houtt.	(Li et al., 2017; Li et al., 2020; Zhang D. et al., 2020)
		Forsythiae Fructus	*Forsythia suspensa* (Thunb.) Vahl
		Isatidis Radix	*Isatis tinctoria* L.
		Bupleuri Radix	*Bupleurum chinense* DC.
		Patriniae Herba	*Patrinia scabiosifolia* Link
		Vervain	*Verbena officinalis* L.
		Phragmitis Rhizoma	*Phragmites australis* subsp. australis
		Glycyrrhizae Radix Et Rhizoma	*Glycyrrhiza inflata* Batalin
Su-He-Xiang	N/A	Styrax	*Liquidambar orientalis* Mill.	(Zhang D. et al., 2020)
		Benzoinum	*Styrax tonkinensis* (Pierre) Craib ex Hartwich
		Borneolum Syntheticumc	*Dryobalanops aromatica* C.F.Gaertn.
		Bubali Cornu*	*Bubalus bubalis* Linnaeus
		Moschus*	*Moschus berezovskii* Flerov
		Santali Albi Lignum	*Santalum album* L.
		Aquilariae Lignum Resinatum	*Aquilaria sinensis* (Lour.) Spreng.
		Aucklandiae Radix	*Aucklandia costus* Falc.
		Cyperi Rhizoma	*Cyperus rotundus* L.
		Olibanum	*Boswellia carteri* Birdw.
		Long Pepper Fruit.	*Piper longum* L.
		Atractylodis Macrocephalae Rhizoma	*Atractylodes macrocephala* Koidz.
		Chebulae Fructus	*Terminalia chebula* Retz.
		Cinnabaris^†^	
An-Gong-Niu-Huang	N/A	Bovis Calculus*	*Bos taurus domesticus* Gmelin	(Zhang D. et al., 2020)
		Bubali Cornu*	*Bubalus bubalis* Linnaeus
		Moschus*	*Moschus berezovskii* Flerov
		Margarita*	
		Cinnabaris^†^	
		Arsenic (II) sulfide^†^	
		Coptidis Rhizoma	*Coptis chinensis* Franch.
		Scutellariae Radix	*Scutellaria baicalensis* Georgi
		Gardeniae Fructus	*Gardenia jasminoides* J.Ellis
		Curcumae Radix	*Curcuma aromatica* Salisb.
		Borneolum Syntheticumc	*Dryobalanops aromatica* C.F.Gaertn.	
Xi-Yan-Ping	N/A	Andrographolide sulfonatesc	*Andrographis paniculata* (Burm.f.) Nees (Burm.f.) Nees	(Zhang D. et al., 2020) (Runfeng et al., 2020; Zhang D. et al., 2020)
Xue-Bi-Jing	LTA4H, 12-LOX, IL2, cPLA2, IL6, RELA, TNF, PTGS2, IL10, NOS2α, CASP3, MAPK1.	Carthami Flos	*Carthamus tinctorius* L.
		Paeoniae Radix Rubra	*Paeonia lactiflora* Pall.
		Chuanxiong Rhizoma	*Conioselinum anthriscoides* ‘Chuanxiong’
		Salvia miltiorrhiza Radix Et Rhizoma	*Salvia miltiorrhiza* Bunge
		Angelicae Sinensis Radix	*Angelica sinensis* (Oliv.) Diels
Re-Du-Ning	COX-2, sEH, IL6, CCL2, CASP3, IL4, MAPK1, RELA, FOS, NOS2, IL1B, CXCL10, MAPK14, EGFR.	Artemisiae Annuae Herba	*Artemisia annua* L.	(Runfeng et al., 2020; Zhang D. et al., 2020)
		Lonicerae Japonicae Flos	*Lonicera Japonica* Thunb.
		Gardeniae Fructus	*Gardenia jasminoides* J.Ellis
Tan-Re-Qing	COX-2, sEH, LTA4H, IL6, IL1B, IL10, MAPK1, IL4, CXCL8, MAPK14, EGFR, CXCL10.	Scutellariae Radix	*Scutellaria baicalensis* Georgi	(Huang et al., 2020; Runfeng et al., 2020; Zhang D. et al., 2020)
		Saigae Tataricae Cornu*	*Saiga tatarica* Linnaeus
		Lonicerae Japonicae Flos	*Lonicera Japonica* Thunb.
		Forsythiae Fructus	*Forsythia suspensa* (Thunb.) Vahl
Xing-Nao-Jing	N/A	Moschus*	*Moschus berezovskii* Flerov
		Borneolum Syntheticum	*Dryobalanops aromatica* C.F.Gaertn.	(Runfeng et al., 2020; Zhang D. et al., 2020)
		Gardeniae Fructus	*Gardenia jasminoides* J.Ellis
		Curcumae Radix	*Curcuma aromatica* Salisb.
Shen-Fu	N/A	Ginseng Radix Et Rhizoma	*Panax ginseng* C.A. Mey.	(Runfeng et al., 2020; Zhang D. et al., 2020)
		Aconiti Lateralis Radix Praeparata	*Aconitum carmichaeli* Debeaux	
Sheng-Mai	IL6, GAPDH, ALB, TNF, MAPK1, MAPK3, TP53, EGFR, CASP3.	Ginseng Radix Et Rhizoma	*Panax ginseng* C.A. Mey.	(Huang et al., 2020; Zhang D. et al., 2020)
		Ophiopogonis Radix	*Ophiopogon japonicus* (Thunb.) Ker Gawl.
Pu-Di-Lan	N/A	Scutellariae Radix	*Scutellaria baicalensis* Georgi	(Kong et al., 2020)
		Traxaci Herba	*Taraxacum mongolicum* Hand. Mazz.
		Corydalis bungeana	*Corydalis bungeana* Turcz.
		Isatidis Radix	*Isatis tinctoria* L.
Yin-Qiao	N/A	Forsythiae Fructus	*Forsythia suspensa* (Thunb.) Vahl	(Huang et al., 2020; Li et al., 2020)
		Lonicerae Japonicae Flos	*Lonicera Japonica* Thunb.
		Platycodonis Radix	*Platycodon grandiflorum* (Jacq.) A. DC.
		Menthae Haplocalycis Herba	*Mentha canadensis* L.
		Phyllostachydis Henonis Folium	*Lophatherum gracile* Brongn.
		Glycyrrhizae Radix Et Rhizoma	*Glycyrrhiza inflata* Batalin
		Schizonepetae Herba	*Nepeta tenuifolia* Benth.
		Sojae Semen Praeparatum	*Glycine max* (L.) Merr.
		Arctii Fructus	*Arctium lappa* L.
Yu-Ping-Feng-San	N/A	Saposhnikoviae Radix	*Saposhnikovia divaricata* (Turcz. ex Ledeb.) Schischk.	(Huang et al., 2020; Li et al., 2020)
		Astragali Radix	*Astragalus mongholicus* Bunge
		Atractylodis Macrocephalae Rhizoma	*Atractylodes macrocephala* Koidz.
Sang-Ju	N/A	Mori Folium	*Morus alba* L.	(Huang et al., 2020; Li et al., 2020)
		Chrysanthemi Flos	*Chrysanthemum × morifolium* (Ramat.) Hemsl.
		Almond	*Prunus armeniaca* L.
		Forsythiae Fructus	*Forsythia suspensa* (Thunb.) Vahl
		Menthae Haplocalycis Herba	*Mentha canadensis* L.
		Platycodonis Radix	*Platycodon grandiflorum* (Jacq.) A. DC.
		Glycyrrhizae Radix Et Rhizoma	*Glycyrrhiza inflata* Batalin
		Phragmitis Rhizoma	*Phragmites australis* subsp. australis
Shuang-Huang-Lian	N/A	Lonicerae Japonicae Flos	*Lonicera Japonica* Thunb.	(Hirano and Murakami, 2020)
		Scutellariae Radix	*Scutellaria baicalensis* Georgi
		Forsythiae Fructus	*Forsythia suspensa* (Thunb.) Vahl
Ma-Xing-Shi-Gan	N/A	Ehedraep Herba	*Ephedra sinica* Stapf	(Li et al., 2020; Yang et al., 2020)
		Almond	*Prunus armeniaca* L.
		Glycyrrhizae Radix Et Rhizoma	*Glycyrrhiza inflata* Batalin
		Gypsum Fibrosum^†^	
Bai-He-Gu-Jin	N/A	Rehmanniae Radix	*Rehmannia glutinosa* (Gaertn.) DC.	(Huang et al., 2020; Li et al., 2020)
		Angelicae Sinensis Radix	*Angelica sinensis* (Oliv.) Diels
		Paeoniae Radix Alba	*Paeonia lactiflora* Pall.
		Glycyrrhizae Radix Et Rhizoma	*Glycyrrhiza inflata* Batalin
		Platycodonis Radix	*Platycodon grandiflorum* (Jacq.) A. DC.
		Scrophulariae Radix	*Scrophularia ningpoensis* Hemsl.
		Fritillaria Thunbergii Bulbus	*Fritillaria thunbergii* Miq.
		Ophiopogonis Radix	*Ophiopogon japonicus* (Thunb.) Ker Gawl.
		Lilii Bulbus	*Lilium lancifolium* Thunb.
Ren-Shen-Bai-Du	N/A	Chinese Thorawax Root.	*Bupleurum scorzonerifolium* Willd.	(Xian et al., 2020)
		Glycyrrhizae Radix Et Rhizoma	*Glycyrrhiza inflata* Batalin
		Incised Notopterygium Rhizome Root	*Hansenia forbesii* (H.Boissieu) Pimenov & Kljuykov
		Doubleteeth Angelicae Root	*Angelica biserrata* (R.H.Shan & C.Q.Yuan) C.Q.Yuan & R.H.Shan
		Chinese Thorawax Root	*Bupleurum scorzonerifolium* Willd.
		Common Hogfennel Root	*Angelica decursiva* (Miq.) Franch. & Sav.
		Chuanxiong Rhizoma	*Conioselinum anthriscoides* ‘Chuanxiong’
		Submature Bitter Orange	*Citrus × aurantium* L.
		Menthae Haplocalycis Herba	*Mentha canadensis* L.
		Poria mushroom^§^	*Poria cocos* (Schw.) Wolf
		Platycodonis Radix	*Platycodon grandiflorum* (Jacq.) A. DC.
		Glycyrrhizae Radix Et Rhizoma	*Glycyrrhiza inflata* Batalin
		Ginger	*Zingiber officinale* Roscoe

^†^Mineral products; ^§^Fungus-derived products; *Animal-derived products; Components without labelling are all plant-derived products.

**Figure 3:**
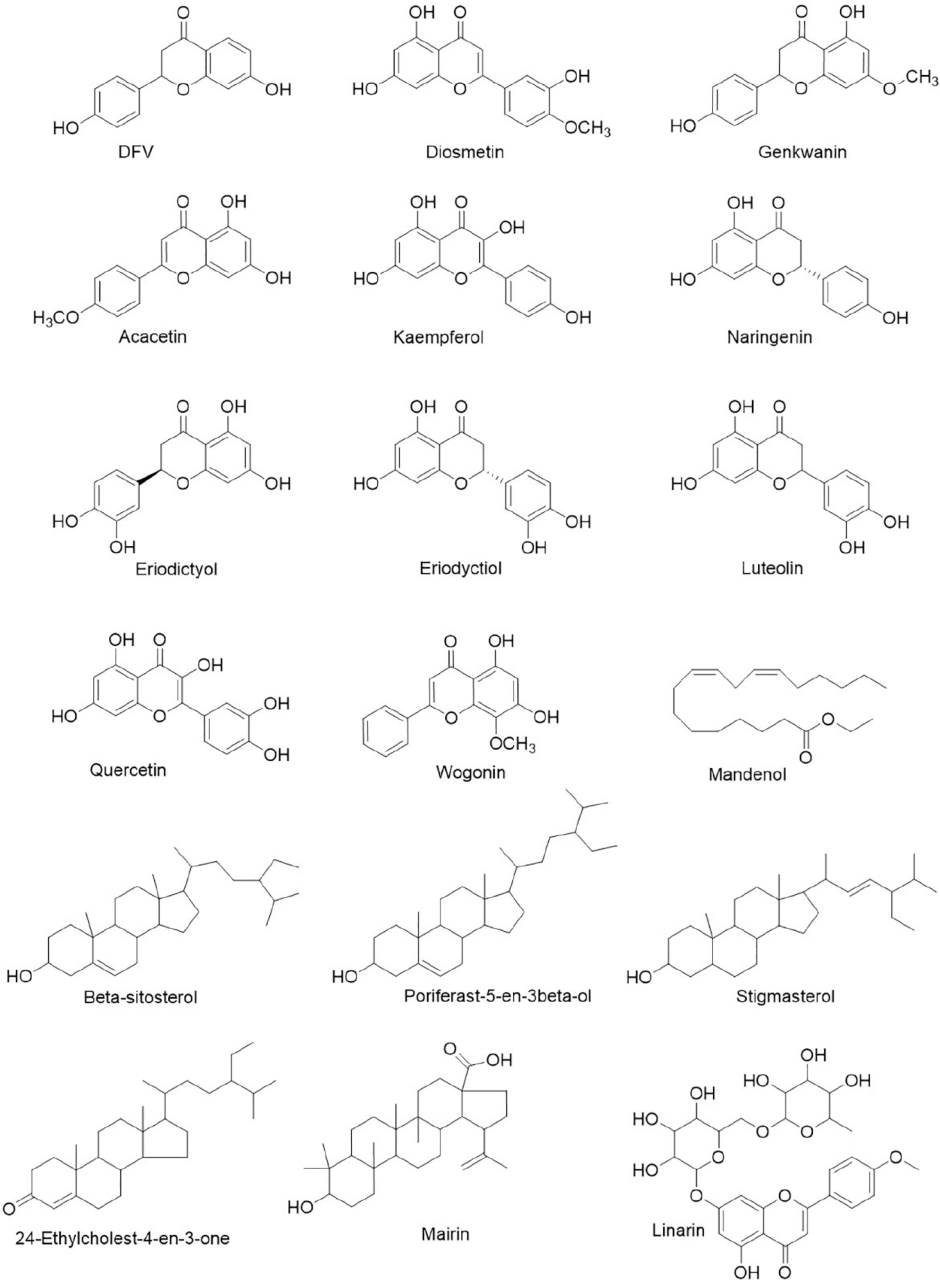
Lead compounds with greatest drug-likeness isolated from herbs for COVID-19.

## Conclusion and Perspective

Since early 2020, numerous pharma companies collaborating with academics or state-sponsored research institute have joined the race of therapy development to combat wildly spreading COVID-19. As of July 2020, a range of various therapeutics has been discovered, from small molecules, neutralizing antibodies, to bioengineered products. Aided with computational chemistry and virtual screening, researchers has established a large library of novel small molecules, showing favorable binding affinity with validated drug targets (Kupferschmidt and Cohen, 2020; Tahir ul Qamar et al., 2020), but the efficacy and toxicity of those lead compounds need further testing in both preclinical models and human subjects. Nevertheless, at this point, it seems optimizing a novel lead for COVID-19 is not a preferred option as typically the whole pipeline of new drug development takes years even if FDA grants expediated approval, and it is unimaginable for the public to endure another prolonged era of economic and public health hardship. As for neutralizing antibody, the situation is also gloomy, because establishing manufacturing infrastructure and managing supply chain of biologics are much more nuanced and demanding than small molecular drug. Thanks to the recent advances in bioengineering, several nanoengineered therapeutics have been designed to treat COVID-19. Zhang et al. recently reported novel nano-sponges made of the plasma membranes derived from human lung epithelial type II cells (Zhang Q. et al., 2020). These nano-sponges display the membrane receptors recognizable to SARS-CoV-2. They showed that, following incubation with their nano-sponges, SARS-CoV-2 lost infectability. Huo et al. produced an array of nanobodies that bind SARS-CoV-2 receptor and block its interaction with ACE2 (Huo et al., 2020). Though such studies open exciting new path for therapeutics discovery, lack of clinical data stops them from becoming relevant in the short terms. Regretfully, to date there is no approved drug for any kinds of human coronavirus infection, including SARS-CoV, MERS-CoV, or SARS-CoV-2.

**Figure 4:**
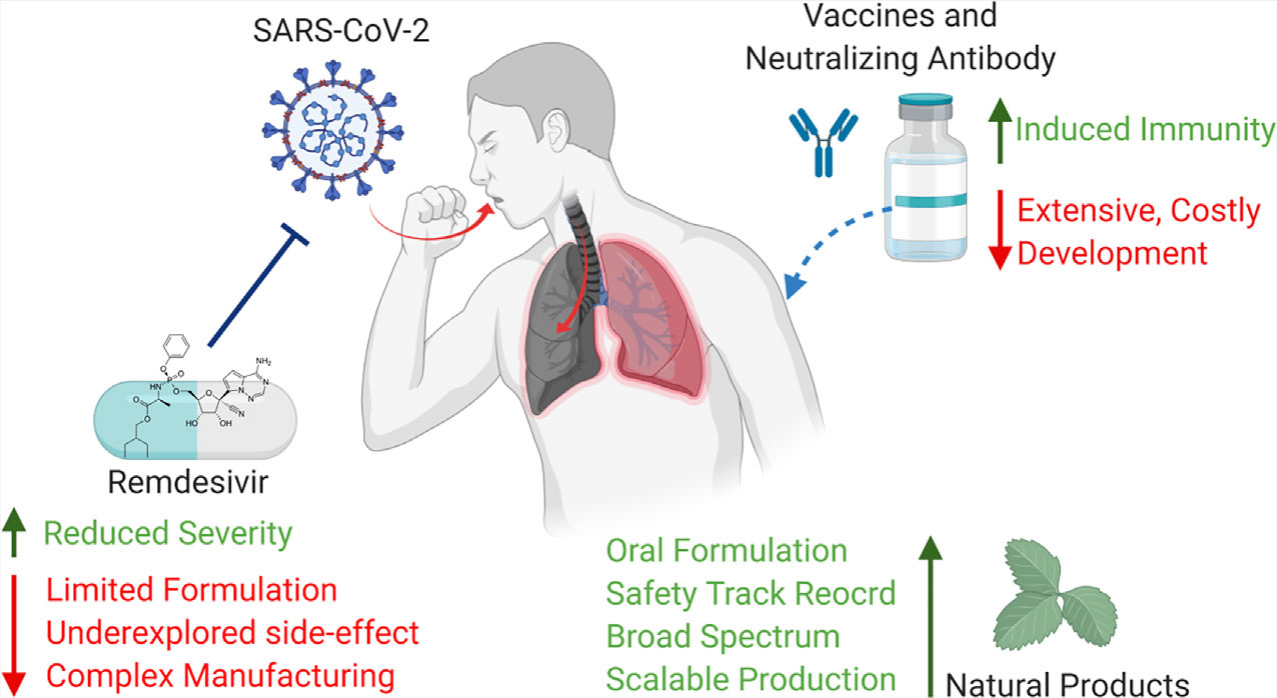
Pros and cons of current prevention of COVID-19 (Created with BioRender.com).

Since the outcome of current therapeutics in severe/critical COVID-19 patients are still debatable, prevention rather than treatment becomes more important to restrain this pandemic. Blocking the entry of SARS-CoV-2 and suppressing infection at initial stage are considered as more practical strategy (**Figure 4**). Vaccine has been historically used to prevent influenza. Today, antibody responses and serum-neutralizing activity are standard parameters used to evaluate the short-term efficacy of vaccine (Jackson et al., 2020), whereas the long-term effectiveness cannot be truly determined until the vaccinated population show acquired immunity against infection when exposed to the virus of interest without intervention. Besides, recent clinical report pointed out that neutralizing antibody level in patients who experienced asymptomatic SARS-CoV-2 infection declined rapidly after recovery (Long et al., 2020), which leads to a concerning question how long vaccination is able to maintain its protection against COVID-19. In terms of pre-exposure/post-exposure prophylaxis, NIH recently launched trials to test the preventive effectiveness of monoclonal antibody. While the trial is ongoing, intravenous (i.v.) injection of antibody in large population brings up a lot of feasibility issues. Even the prophylactic efficacy of remdesivir was proven to be better than its therapeutic efficacy in rhesus macaque model, current administration route of remdesivir is limited to i.v. infusion that restricts its use in non-hospitalized population (Administration).

Natural products and herbal medicine have long track record to treat respiratory infection and many have been approved as drugs, over-the-counter nutrition or food additives. Those products generally have satisfactory safety profiles. The minimal toxicity makes natural product and herbal medicines ideal prophylactic candidates for long-term use. Based on recent *in silico* results, an array of natural products has been found highly potent in blocking enzyme function and membrane receptors of human coronavirus. Moderate dosing of such bioactive compounds may prevent or at least slow down SARS-CoV-2 infection process. In addition, the progression of COVID-19 is featured with uncontrolled inflammation, like cytokine release syndrome, so anti-inflammatory herbs will be a potential tool to suppress such fatal symptom. The stability of natural products and herbal medicines in human gastrointestinal tract is barely an issue. The low pH in gastric environment, digestive enzymes, and gut microbiome have less impact on the bioavailability of natural products and herbs compared to antibody and other prophylactics. This advantage makes oral dosing rather than IV administration possible. In terms of availability, the ease of production expansion realizes the mass deployment of herbal medicines to big population, while the large-scale synthesis of monoclonal antibody and remdesivir is incredibly challenging. In this day and age, a safe, effective and stable form of oral dosage prophylactics will be a strong asset for us to overcome COVID-19 pandemic.

## Author Contributions

Conceptualization: GT, JC, and JH. Writing—original draft preparation: GT, JL, and JC. Writing—review and editing: GT, JL, JC, and JH. Visualization: ZH, JH. Supervision: GT and JH. Funding acquisition: J-xC and JH. All authors contributed to the article and approved the submitted version.

## Funding

This work is supported by the Huang Zhendong Research Fund for Traditional Chinese Medicine of Jinan University (No. 201911).

## Conflict of Interest

The authors declare that the research was conducted in the absence of any commercial or financial relationships that could be construed as a potential conflict of interest.

## Abbreviations

ACE2, angiotensin-converting enzyme 2; ADMET, absorption, distribution, metabolism, excretion and toxicity; AKTI, Akt inhibitor; ALB, albumin; AR, androgen receptor; BAX, Bcl-2 Assaciated X protein; BCL2, B-cell lymphoma-2; BCL2L, bcl2-like gene; CASP3, caspases-3; CCL2, chemokine (C-C motif) ligand 2; COX-2, cyclooxygenase-2; COVID-19, coronavirus disease 2019; CoVs, coronaviruses; cPLA2, cytosolic Phospholipase A2; cPLA2α, cytosolic Phospholipase A2α; CXCL10, CXC motif chemokine 10; CXCL8, CXC motif chemokine 8; DPP4, Dipeptidyl peptidase-4; EGFR, Epidermal Growth Factor Receptor; ESR1, Estrogen Receptor 1; F10, Coagulation Factor X; FDA, food and drug administration; FRET, fluorescence resonance energy transfer; HCoV-229E, human coronavirus 229E; FOS, Fos proto-oncogene; GAPDH, glyceraldehyde-3-phosphate dehydrogenase; HCoV-HKU1, human coronavirus HKU1; HCoV-NL63, human coronavirus NL63; HCoV-OC43, human coronavirus OC43; HCS, high-content screening; HSPA5, heat shock protein A5; HSP90AA1, heat shock protein 90 kDa alpha, class A member 1; HSP90AB1, heat shock protein 90 kDa alpha, class B member 1; IL10, interleukin 10; IL-1β, interleukin 1β; IL2, interleukin 2; IL4, interleukin 4; IL6, interleukin 6; JUN, Jun proto-oncogene; LTA4H, leukotriene A4 hydrolase; MAPK1, mitogen-activated protein kinase 1; MAPK14, mitogen-activated protein kinase 14; MAPK3, mitogen-activated protein kinase 3; MAPK8, mitogen-activated protein kinase 8; MERS-CoV, middle east respiratory syndrome-related coronavirus; mPGES-1, Microsomal prostaglandin E synthase-1; NAb, neutralizing antibody; NOS2, Nitric oxide synthase 2; NOS2α, Nitric oxide synthase 2α; NHC, national health commission of the P.R. China; NHS, national health service; NHSBT, blood and transplant service; NIAID, national institute of allergy and infectious diseases; NTP, nucleotide triphosphate; PIK3CG, Phosphatidylinositol-4; PTGS1, Prostaglandin-Endoperoxide Synthase 1; PTGS2, Prostaglandin-Endoperoxide Synthase 2; PLpro, papain-like protease; RBD, receptor-binding domain; RdRp, RNA-dependent RNA polymerase; RELA, RELA proto-oncogene; S protein, Spike protein; SARS-CoV, severe acute respiratory syndrome coronavirus; SARS-CoV-2, severe acute respiratory syndrome coronavirus 2; SBDβ, substrate-binding domain β; sEH, soluble epoxide hydrolase; TMPRSS2, transmembrane protease; TNF, Tumor necrosis factor; TP53, tumor protein p53; WHO, world health organization; 3CLpro, 3-chymotrypsin-like protease; 5-LOX, 5-liopoxygenase; 12-LOX, 12-liopoxygenase.
